# Association between migraine and the risk of vascular dementia: A nationwide longitudinal study in South Korea

**DOI:** 10.1371/journal.pone.0300379

**Published:** 2024-04-17

**Authors:** Hyomin Shin, Woo Seok Ha, Jaeho Kim, Sang Hyun Park, Kyungdo Han, Min Seok Baek

**Affiliations:** 1 Department of Neurology, Wonju Severance Christian Hospital, Yonsei University Wonju College of Medicine, Wonju, South Korea; 2 Department of Neurology, Gangwon-do Wonju Medical Center, Wonju, South Korea; 3 Department of Neurology, Severance Hospital, Yonsei University College of Medicine, Seoul, South Korea; 4 Department of Neurology, Dongtan Sacred Heart Hospital, Hallym University College of Medicine, Hwaseong-si, Gyeonggi-do, South Korea; 5 Department of Statistics and Actuarial Science, Soongsil University, Seoul, South Korea; 6 Research Institute of Metabolism and Inflammation, Yonsei University Wonju College of Medicine, Wonju, South Korea; University of Caytania, ITALY

## Abstract

**Objective:**

We aimed to examine the potential association between migraine and vascular dementia (VaD) using a nationwide population database.

**Background:**

Migraine and VaD showed similar structural and functional changes in pathophysiology process and shared common risk factors, However, whether migraine prevalence increases VaD incidence remains controversial.

**Methods:**

This retrospective population-based cohort study used the medical records from the Korean National Health Insurance System database. Migraine (G43) was defined by using the Tenth Revision of the International Classification of Diseases code. More than two migraine diagnoses at least 3 months apart were defined as “chronic migraine”. Cox proportional hazards model estimated hazard ratios (HRs) of VaD for group comparisons.

**Results:**

We included 212,836 patients with migraine and 5,863,348 individuals without migraine. During 10 years of follow-up, 3,914 (1.8%) and 60,258 (1.0%) patients with and without migraine, respectively, were newly diagnosed with VaD. After adjustment, patients with migraine showed a 1.21-fold higher risk of VaD than those without migraine (HR = 1.21; 95% confidence interval (CI): 1.17–1.25). Patients with chronic migraine showed a higher cumulative incidence of VaD than those with episodic migraine. The adjusted HR for the VaD incidence with migraine was higher in: (1) patients aged <65 years; (2) women; (3) patients without hypertension, diabetes, or atrial fibrillation; and (4) non-smokers.

**Conclusion:**

Migraine is associated with an increased risk of VaD, particularly in chronic migraine patients. Incidence of VaD in the setting of migraine may have distinct pathophysiology from that of VaD with traditional cardiovascular risks.

## Introduction

Vascular dementia (VaD) is one of the predominant causes of dementia, accounting for 15% of all dementia cases [1, 2]. VaD identifies a cognitively impaired status with loss of independent daily function, and it comprises the umbrella term, vascular cognitive impairment (VCI), together with mild VCI and mixed dementia [3]. VaD shares common risk factors with cardiovascular diseases, such as hypertension, diabetes, and dyslipidemia [4, 5]. Multiple lacunes, strategic infarcts, and white matter abnormalities in imaging studies are common findings in patients with VaD [4, 6]. Covert cerebral small vessel disease in persons without overt neurological symptoms also increases the risk of dementia [7].

Migraine is a complex brain disorder that involves vascular dysfunction and short-term nerve activation, as well as alterations in hormonal regulation and neuromodulation, the latter of which can induce chronic physiological and anatomical changes in the brain [8–11]. Previous neuroimaging studies have revealed increased white matter abnormalities in patients with migraine, with higher risk in those with more frequent and severe attacks [12, 13]. Silent infarct- like lesions have also been reported in patients with migraine, with higher risk of occurrence in the posterior circulation territory [14, 15]. Physiologically, migraine involves cortical spreading depression with altered cortical excitability [16, 17]. Transcranial magnetic stimulation studies in the patients with VCI also showed enhanced excitability and plasticity which is considered as a compensatory mechanism [18].

While traditional cardiovascular (CV) risk factors such as hypertension, diabetes, and dyslipidemia increase the risk of VaD [4, 5], the effect of CV risks on migraine is controversial. The Genetic Epidemiology of Migraine (GEM) study has shown that patients with migraine with aura are more likely to have greater CV risks such as hypertension and unfavorable cholesterol profile [19], although other studies have reported that higher CV risks are not associated with active or incident migraine [20, 21].

Previous study results on migraine and cognitive function have been contradictory. Some studies suggest that those who experience migraine have cognitive deficits in memory and attention [22, 23], whereas other studies have not found any statistically significant difference in cognitive function between patients with migraine and healthy controls [24]. In one cross-sectional study, a history of severe headache was associated with an increased volume of brain white matter hyperintensities, but these abnormalities were not related to cognitive impairment [25]. Longitudinal studies with a sufficient sample size are necessary to comprehensively examine the impact of migraine on cognitive phenotype or susceptibility to developing VaD.

In the current study, we investigated the relationship between migraine and VaD, which presents the advantage of a large sample size and long-term data availability through the use of national insurance claims data. We hypothesized that patients with migraine would have higher incidence of VaD than those without migraine. Additionally, the effect of migraine subtypes based on the existence of aura and chronification of migraine was examined in order to better understand the role of migraine in VaD development.

## Methods

### Data source

We conducted a retrospective analysis of a nationwide population-based cohort utilizing the Korean National Health Insurance Service (NHIS) database. The NHIS is a compulsory insurance system that provides medical services for the entire Korean population. The NHIS database contains diagnostic codes, prescription records, insurance types, and population characteristics, with a unique anonymous number assigned to each patient. The NHIS also provides a free general medical check-up program for all receivers over 40 years of age, with screenings occurring every 2 years. Health Insurance Review and Assessment Service ensures quality assurance, assesses healthcare efficacy and evaluates medical information [26]. This database is managed by the “Big Data Steering Department” of the NHIS for research objective and includes data on the entire Korean population. Data access for the study was conducted in September 2022.

The health examination includes a self-reported health behavior questionnaire, as well as clinician-assessed medical history, laboratory tests, and measured anthropometric data. This study obtained ethical approval from the NHIS Inquiry Commission and the Institutional Review Board of Wonju Severance Christian Hospital (CR322303) and the research protocol was aligned with the principles of the Declaration of Helsinki and its subsequent revisions. The necessity for informed consent was officially exempted because of the secondary analytic study design and the use of de-identified participant information.

Diagnoses of medical conditions, including hypertension, diabetes, dyslipidemia, congestive heart failure, myocardial infarction, stroke, atrial fibrillation, and depression were based on ICD-10 codes (S1 Table). Estimated glomerular filtration rate (eGFR), smoking status (never, former, or current), drinking status (none, mild to moderate, or heavy), and physical activity were acquired from blood tests as well as the survey administered as part of the National Health Screening Program.

Heavy drinking was defined as consumption of ≥30 g of alcohol per day. To meet the criteria of regular exercise, participants needed to perform vigorous physical activity at least three times per week, or engage in moderate or light physical activity at least five times per week, in alignment with the guidelines outlined by the American College of Sports Medicine [27]. Anthropometric measurements, including height, weight, and waist circumference, were also assessed. Body mass index (BMI) was computed by dividing weight by the square of height in meters.

### Study population

We selected individuals who engaged in the National Health Screening Program in 2009. Of these, participants aged ≥40 years were selected for the present study. To evaluate the prevalence and incidence of migraine within the NHIS database, medical records from the NHIS database were investigated, covering the period from 2002 to 2019. Patients with migraine were defined based on the Tenth Revision of International Classification of Diseases code (ICD-10) G43 in their medical records within 12 months of enrollment. Individuals without migraine were those who had no record of migraine listed in their medical records from 2002 to 2009. Migraine with aura was defined using ICD-10 code G431. Individuals were identified as having chronic migraine if migraine headache occurred at least twice in a greater than 3-month interval within 1 year, while the remaining individuals with migraine were considered to have episodic migraine.

**Fig 1:**
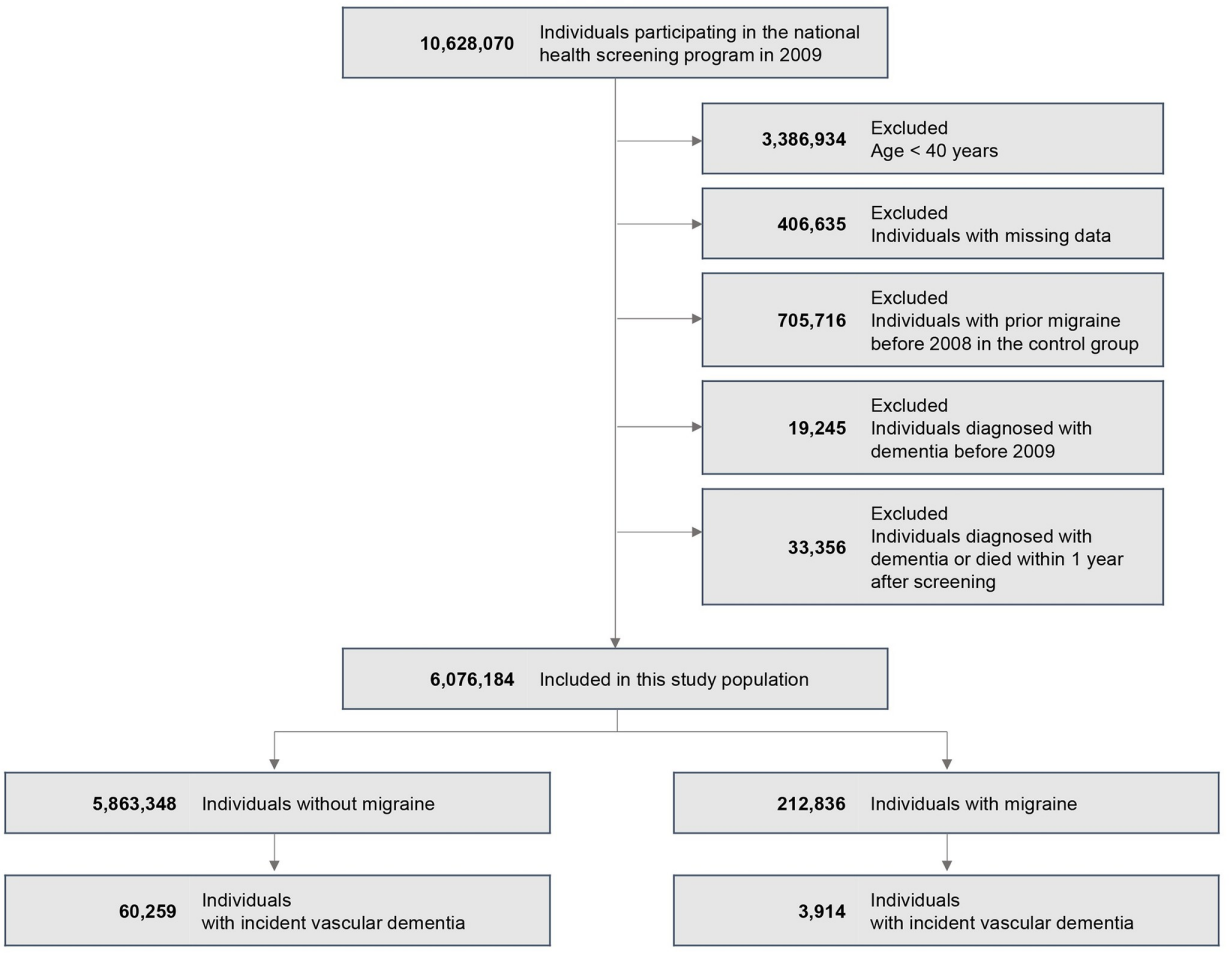
Study flowchart.

VaD was identified using the ICD-10 code F01, along with patients’ prescription records for anti-dementia medication. Eligible medications were limited to acetylcholinesterase inhibitors (rivastigmine, galantamine, donepezil) or N-methyl-D-aspartate (NMDA)-receptor antagonist (memantine). The date of dementia diagnosis was determined as the point at which the prescription of medication and the registration of a dementia code coincided. The participant with previous diagnosis of Alzheimer’s disease defined by having the code F00 (Dementia in Alzheimer disease) or G30 (Alzheimer disease), and other dementia (F02, F03, or G31) in the NHIS database were excluded. In Korea, to file expense claims for the prescription of the aforementioned medication for dementia treatment, physicians are required to document evidence of cognitive dysfunction according to the National Health Insurance Reimbursement criteria: a Mini-Mental State Examination score of ≤26 and either a Clinical Dementia Rating of ≥1 or a Global Deterioration Scale score of ≥3. The diagnostic standard for dementia using ICD-10 codes have been detailed in prior investigations [28–30]. Participants with a previous VaD diagnosis and those who developed VaD or died within 12 months of enrollment were also excluded. To reduce the likelihood of reverse causation, 12 months was used for the wash-out period. The study flowchart is depicted in Fig 1.

### Statistical analysis

The primary study outcome was the development of VaD. Each participant was followed up from the screening date in 2009 to December 31, 2019, unless censored due to death. Hazard ratios (HR) were calculated using the Cox proportional hazards model, with their associated 95% confidence intervals (CI) to assess the risk of VaD in individuals with migraine compared with those without migraine. Four models were used. Model 1 was a crude analysis, without any adjustment. Adjustments for age and sex were made in Model 2. Model 3 was adjusted for age, sex, comorbidities (hypertension, diabetes, dyslipidemia, congestive heart failure, myocardial infarction, and stroke), lifestyle (smoking status, drinking status, and regular exercise), eGFR, and BMI. Model 4 was adjusted for covariates in Model3 and diagnoses of atrial fibrillation and depression. Subgroup analyses using a multivariate Cox regression hazard model were performed to evaluate the influence of preexisting comorbidities or demographic attributes on the risk of VaD in the patients with migraines. The SAS statistical software (version 9.2; SAS Institute, Cary, NC, USA) was used for all statistical analyses. A two-sided p-value of <0.05 was considered statistically significant.

## Results

### Population characteristics

A total of 212,836 patients with migraine and 5,863,348 individuals without migraine were included in this study. Patients with migraine (56.5 years) were older than those without migraine (54.0 years), and the proportion of women was higher among patients with migraine than in those without migraine (Table 1). Diabetes was more prevalent in individuals without migraine, whereas hypertension, dyslipidemia, congestive heart disease, myocardial infarction and stroke were more prevalent in the migraine group (Table 1). The proportion of individuals who never smoked and were non-drinkers was higher among the migraine group, whereas the proportion of those who engaged in regular exercise was higher among those without migraine (Table 1).

**Table 1:** Demographic characteristics.

	No. (%)		p-value
	Without migraine	With Migraine	
**N**	5,863,348	212,836	
**Age, mean (SD), y**	53.98 (10.3)	56.49 (10.9)	<0.001
**Women (%)**	2,734,541 (46.6)	153,766 (72.3)	<0.001
**Comorbidities (%)**			
**Hypertension**	1,996,596 (34.1)	90,278 (42.4)	<0.001
**Diabetes**	680214 (11.6)	23,679 (11.1)	<0.001
**Dyslipidemia**	1,238,819 (21.1)	59,135 (27.8)	<0.001
**Myocardial infarction**	28,983 (0.5)	1,751 (0.8)	<0.001
**Congestive heart failure**	41,904 (0.7)	3,238 (1.5)	<0.001
**Stroke**	106,465 (1.8)	12,216 (5.7)	<0.001
**Atrial fibrillation**	36,980 (0.63)	2,003 (0.94)	<0.001
**Depression**	182,775 (3.12)	28,197 (13.25)	<0.001
**BMI, kg/m**^**2**^ **(SD)**	23.9 (3.03)	24.0 (3.1)	<0.001
**Waist circumference, cm (SD)**	81.3 (8.58)	80.5 (8.68)	<0.001
**eGFR, mL/min/1.73 m**^**2**^ **(SD)**	85.0 (38.2)	83.7 (32.0)	<0.001
**Lowest income quartile (%)**	1,183,345 (20.2)	45,368 (21.3)	<0.001
**Smoking (%)**			<0.001
**Never smoker**	3,588,441 (61.2)	169,257 (79.5)	
**Ex-smoker**	961,144 (16.4)	20,548 (9.7)	
**Current smoker**	1,313,763 (22.4)	23,031 (10.8)	
**Drinking (%)**			<0.001
**Non-drinker**	3,314,642 (56.5)	157,915 (74.2)	
**Mild to moderate drinker (<30g/d)**	2,091,280 (35.7)	47,406 (22.3)	
**Heavy drinker (≥30 g/d)**	457,426 (7.8)	7,515 (3.5)	
**Regular exercise (%)**	1,187,550 (20.3)	38,955 (18.3)	<0.001

Abbreviations: BMI, body mass index (calculated as weight in kilograms divided by height in meters squared); eGFR, estimated glomerular filtration rate; SD, standard deviation.

### VaD incidence and underlying characteristics in patients with migraine

**Fig 2:**
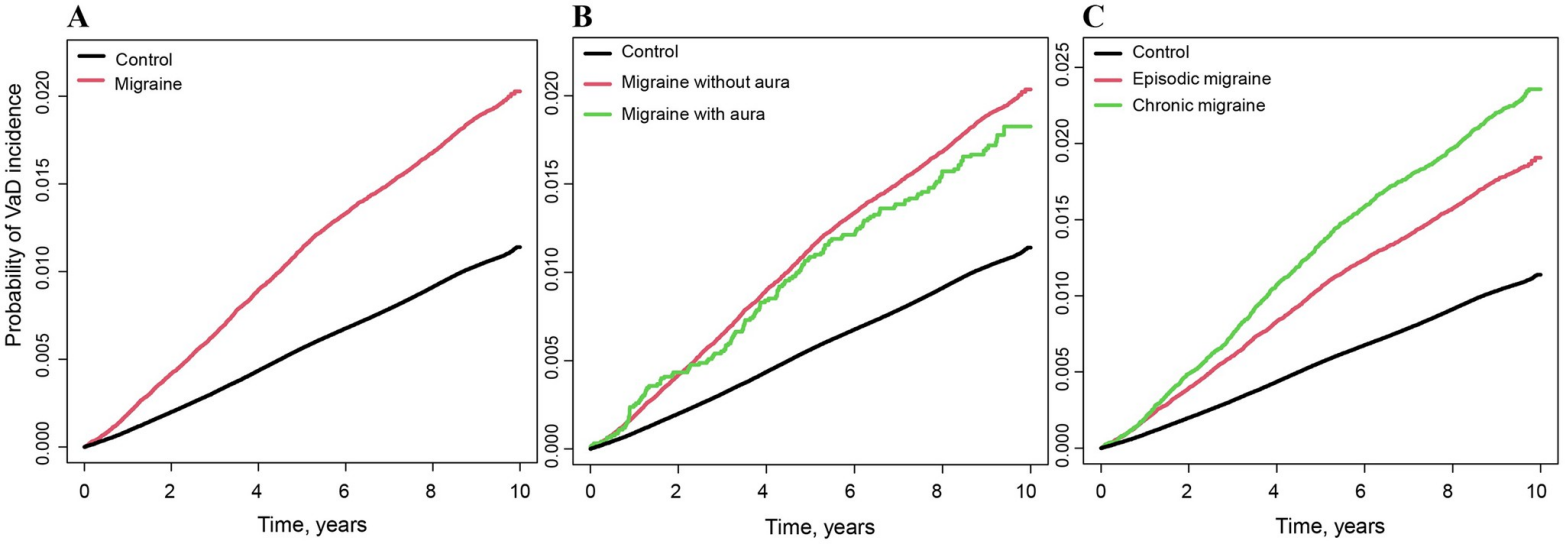
Kaplan-Meier curves of the incidence of vascular dementia in individuals with migraine. (A) Migraine versus no migraine. (B) Migraine with aura versus migraine without aura versus no migraine. (C) Chronic migraine versus episodic migraine versus no migraine.

During the 10 years of follow-up (54,218,426 person-years; median, 9.0 years), 64,173 participants (1.1%) were diagnosed with incident VaD. In patients with migraine, 1.8% (3,914/212,836) were diagnosed with VaD, while in individuals without migraine, 1.0% (60,259/5,863,348) were diagnosed with VaD; this suggests an increased risk of VaD in patients with migraine (Table 2). Moreover, after adjustment for covariates, individuals with migraine showed a 1.21-fold higher risk of incident VaD than those without migraine (adjusted HR = 1.21; 95% CI: 1.17–1.25, Table 2). In a log-rank test, the cumulative incidence of VaD was found to be significantly higher in the migraine group (log-rank P <0.001, Fig 2A). Additionally, patients with migraine with aura were 1.25-fold more likely to develop VaD than those without migraine (adjusted HR = 1.25; 95% CI: 1.07–1.46, Table 2), while patients with migraine without aura showed a 1.21-fold higher risk of VaD after covariate adjustment (adjusted HR = 1.21; 95% CI: 1.17–1.25, Table 2). However, the cumulative incidence of VaD in both groups did not differ in the log-rank test (Fig 2B). Patients with chronic migraine were 1.33-fold more likely to develop VaD than those without migraine (adjusted HR = 1.33; 95% CI: 1.26–1.41, Table 2), while patients with episodic migraine showed a 1.16-fold higher probability of VaD diagnosis after adjustment (adjusted HR = 1.16; 95% CI: 1.12–1.21, Table 2). The cumulative incidence of VaD was also higher in patients with chronic migraine than in those with episodic migraine (log-rank P <0.001, Fig 2C).

**Table 2:** Cox proportional hazard regression analysis of the risk of vascular dementia in individuals with several types of migraine.

Group	No.	VaD No.	Person-years	Incidence rate / 1000 person-y	HR (95% CI)			
					Model 1^a^	Model 2^b^	Model 3^c^	Model 4^d^
**Control**	5,863,348	60,259	52,640,741	1.145	1 (Ref.)	1 (Ref.)	1 (Ref.)	1 (Ref.)
**Migraine**	212,836	3,914	1,877,685	2.084	1.82 (1.77,1.88)	1.36 (1.32,1.41)	1.27 (1.23,1.31)	1.21 (1.17, 1.25)
**Without aura**	203,517	3,757	1,794,787	2.093	1.83 (1.77,1.89)	1.36 (1.32,1.41)	1.27 (1.22,1.31)	1.21 (1.17,1.25)
**With aura**	9,319	157	82,898	1.894	1.66 (1.42,1.94)	1.42 (1.21,1.66)	1.31 (1.12,1.53)	1.25 (1.07,1.46)
**Episodic**	154,585	2,669	1,368,355	1.951	1.71 (1.64,1.77)	1.29 (1.24,1.34)	1.21 (1.16,1.26)	1.16 (1.12,1.21)
**Chronic**	58,251	1,245	509,330	2.444	2.14 (2.02,2.26)	1.54 (1.46,1.63)	1.41 (1.34,1.50)	1.33 (1.26,1.41)

Abbreviations: BMI, body mass index; CI, confidence interval; eGFR, estimated glomerular filtration rate; HR, hazard ratio.

^a^Unadjusted.

^b^Adjusted for age and sex.

^c^Adjusted for age, sex, comorbidities (hypertension, diabetes, dyslipidemia, myocardial infarction, congestive heart failure, and stroke), eGFR, BMI, and lifestyle (smoking status, drinking status, and regular exercise).

^d^Adjusted for age, sex, comorbidities (hypertension, diabetes, dyslipidemia, myocardial infarction, congestive heart failure, stroke, atrial fibrillation, and depression), eGFR, BMI, and lifestyle (smoking status, drinking status, and regular exercise).

### Association between VaD incidence and migraine: Underlying characteristics

The risk of incident VaD was analyzed with stratification according to age, sex, underlying comorbidities, BMI, and current smoking status (Table 3). The incidence of VaD tended to be higher in older participants (aged ≥65 years) and in those with underlying hypertension, diabetes, or dyslipidemia (Table 3). However, the HR in patients with migraine, as compared with the controls in each subgroup, was relatively higher in the younger age group (adjusted HR = 1.29; 95% CI: 1.20–1.38 in those aged <65 and adjusted HR = 1.18; 95% CI: 1.14–1.23 in those aged ≥65, P for interaction = 0.031), in women (adjusted HR = 1.26; 95% CI: 1.21–1.31 in women and adjusted HR = 1.09; 95% CI: 1.03–1.16 in men, P for interaction < 0.001), and in those without underlying hypertension (adjusted HR = 1.33; 95% CI: 1.25–1.40 in patients without hypertension and adjusted HR = 1.16; 95% CI: 1.12–1.21 in patients with hypertension, P for interaction <0.001), and diabetes (adjusted HR = 1.24; 95% CI: 1.20–1.29 in patients without diabetes and adjusted HR = 1.11; 95% CI: 1.04–1.19 in patients with diabetes, P for interaction = 0.006), and in non-smokers (adjusted HR = 1.29; 95% CI: 1.24–1.33 in non-smokers and adjusted HR = 1.10; 95% CI: 0.99–1.23 in smokers, P for interaction = 0.008).

**Table 3:** Multivariable Cox proportional hazards regression analysis of the risk of vascular dementia in individuals with migraine.

		Group	Participants(n)	VaD diagnosis(n)	Person-years	Incidence rate (/ 1000py)	Model 4^a^, HR (95% CI)	P for interaction
**Age**	**Age <65**	Control	4,821,001	16,966	44,383,171	0.382	1 (Ref.)	0.031
		Migraine	158,240	866	1,459,465	0.593	1.29 (1.20,1.38)	
	**Age ≥65**	Control	1,042,347	43,293	8,257,569	5.243	1 (Ref.)	
		Migraine	54,596	3,048	418,219	7.288	1.18 (1.14,1.23)	
**Sex**	**Men**	Control	3,128,807	29,694	27,924,657	1.063	1 (Ref.)	<0.001
		Migraine	59,070	996	512,588	1.943	1.09 (1.03,1.16)	
	**Women**	Control	2,734,541	30,565	24,716,083	1.237	1 (Ref.)	
		Migraine	153,766	2,918	1,365,095	2.138	1.26 (1.21,1.31)	
**Hypertension**	**No**	Control	3,866,752	22,269	35,259,722	0.632	1 (Ref.)	<0.001
		Migraine	122,558	1,284	1,109,718	1.157	1.33 (1.25,1.40)	
	**Yes**	Control	1,996,596	37,990	17,381,018	2.186	1 (Ref.)	
		Migraine	90,278	2,630	767,966	3.425	1.16 (1.12,1.21)	
**Diabetes**	**No**	Control	5,183,134	45,488	46,823,813	0.971	1 (Ref.)	0.006
		Migraine	189,157	3,064	1,683,329	1.820	1.24 (1.20,1.29)	
	**Yes**	Control	680,214	14,771	5,816,927	2.539	1 (Ref.)	
		Migraine	23,679	850	194,355	4.373	1.11 (1.04,1.19)	
**Dyslipidemia**	**No**	Control	4,624,529	42,516	41,617,455	1.022	1 (Ref.)	0.059
		Migraine	153,701	2,433	1,363,593	1.784	1.18 (1.13,1.23)	
	**Yes**	Control	1,238,819	17,743	11,023,285	1.610	1 (Ref.)	
		Migraine	59,135	1,481	514,091	2.881	1.26 (1.20,1.33)	
**Atrial fibrillation**	**No**	Control	5,826,368	58,824	52,346,480	1.124	1 (Ref.)	0.028
		Migraine	210,833	3,826	1,861,701	2.055	1.22 (1.18,1.26)	
	**Yes**	Control	36,980	1,435	294,260	4.877	1 (Ref.)	
		Migraine	2,003	88	15,982	5.503	0.96 (0.77,1.18)	
**Depression**	**No**	Control	5,680,573	55,521	51,085,175	1.087	1 (Ref.)	0.043
		Migraine	184,639	3,065	1,638,164	1.871	1.23 (1.19,1.28)	
	**Yes**	Control	182,775	4,738	1,555,566	3.046	1 (Ref.)	
		Migraine	28,197	849	239,520	3.545	1.13 (1.05,1.22)	
**BMI**	**BMI <25**	Control	3,822,407	39,390	34,228,948	1.151	1 (Ref.)	0.519
		Migraine	137,202	2,501	1,207,476	2.071	1.20 (1.15,1.25)	
	**BMI ≥25**	Control	2,040,941	20,869	18,411,792	1.133	1 (Ref.)	
		Migraine	75,634	1,413	670,207	2.108	1.23 (1.16,1.30)	
**Current smoker**	**No**	Control	4,549,585	49,305	40,910,768	1.205	1 (Ref.)	0.014
		Migraine	189,805	3,568	1,676,906	2.128	1.23 (1.19,1.27)	
	**Yes**	Control	1,313,763	10,954	11,729,972	0.934	1 (Ref.)	
		Migraine	23,031	346	200,777	1.723	1.07 (0.96,1.18)	

Abbreviations: BMI, body mass index; CI, confidence interval, eGFR, estimated glomerular filtration rate; HR, hazard ratio; VaD, vascular dementia.

^a^Adjusted for age, sex, comorbidities (hypertension, diabetes, dyslipidemia, myocardial infarction, congestive heart failure, stroke, atrial fibrillation, and depression), eGFR, BMI, and lifestyle (smoking status, drinking status, and regular exercise).

### Subgroup analysis

We conducted a risk-stratified analysis to further assess the impact of migraine subtypes based on the presence of aura in specific patient subgroups. HRs of VaD were calculated in subgroups stratified according to participants’ age, sex, BMI, smoking status, and presence of underlying comorbidities. Significant interactions were observed according to age, sex, smoking status, and underlying hypertension, diabetes, or dyslipidemia (P for interaction < 0.05; S2 Table).

## Discussion

We found that migraine increased the risk of incident VaD in this large-scale, nationwide, population-based longitudinal cohort study examining >6 million people over a mean follow-up period of 9 years. The effect of migraine was relatively greater in younger patients, as well as in women, non-smokers, and those without hypertension or diabetes. Patients with chronic migraine exhibited a heightened vulnerability to developing VaD than those with episodic migraine based on age, sex, and underlying comorbidities.

We observed a 1.21-fold higher risk of VaD among patients with migraine, and the association remained statistically significant in both men and women after covariate adjustment. Previous studies showed inconsistent findings for the relationship between migraine and VaD. One study reported a higher incidence of VaD only in female patients with migraine (adjusted HR = 3.1; 95% CI: 1.0–8.1 in women and adjusted HR = 2.4; 95% CI: 0.6–10.4 in men) [31], while other studies did not show statistically significant findings for the incidence of VaD in patients with migraine [32–34]. In Danish twin cohort study, cognitive test results between patients with migraine and those without migraine showed no statistical difference [35], and another longitudinal study showed no effects of migraine on score changes of mini mental status examination between patients with migraine and those without migraine [36]. Meta-analysis results pooling multiple studies, however, showed that migraine was associated with an increased risk of VaD (relative risk = 1.85; 95% CI: 1.22–2.81) [37]. The reason for these inconsistent findings may be due to varying sample sizes and follow-up durations across studies. In addition, the distribution of sex, age, and underlying comorbidities in the study populations, and the age-dependent increasing risk of VaD may have impacted comparative results as well.

The current study demonstrated that the effect of migraine on the development of VaD was higher in women. This result was concurrent with those of previous studies [31, 32]. The underlying mechanism remains unknown. However, increased prevalence of white matter hyperintensities in women suffering from migraine [9], as well as sex-dependent structural and functional alterations, may have affected the results [38, 39]. The higher proportion of women with chronic migraine enrolled in this study may also have affected the results.

Previous studies have reported that traditional CV risk factors such as hypertension, diabetes and smoking are associated with increased risk of VaD [5]. Alteration in the cerebral hemodynamics potentially occurred with the migraine is also associated with migrainous infarction [40], and executive dysfunction in the patient with vascular depression [41]. Although the incidence of VaD was higher in patients with CV risks in this study, the HR of VaD in patients with migraine, as compared with that in the controls in each subgroup, was unexpectedly higher in patients without hypertension or diabetes. These results are consistent with those of previous studies on CV risks on migraine, which have shown that higher traditional CV risks are not associated with active or incident migraine [20, 21], and the risk of migraine-associated stroke was greater in women without CV risks such as hypertension, diabetes, and MI [42], despite an overall increased risk of stroke associated with migraine [43]. Our results suggest that VaD in the setting of migraine has distinct pathophysiology from VaD with traditional CV risks.

Several restrictions were identified in this study. First, this study is based on the retrospective analysis, which is particularly susceptible to the effects of reverse causality bias. To minimize the bias, we limited the participants to the newly diagnosed migraine patients in 2009 and set the wash-out period for the cumulative incidence analysis. Second, the diagnosis of migraine and the determination of migraine type were based solely on ICD-10 codes as derived from the NHIS database. We note that, in this study, the 1-year migraine prevalence rate in 2009 was 3.1%. However, a nationwide, cross-sectional survey conducted in Korea in 2009 following the International Classification of Headache Disorders, Second Edition criteria revealed a 1-year migraine prevalence of 6.0% [44]. Considering that only individuals who underwent medical consultations for migraine were included in our study, we believe that the diagnosis of migraine in this study was underestimated, which could affect the statistical relationships between variables by introducing measurement errors [45]. Moreover, the proportion of patients with migraine with aura among all patients with migraine was low (4.4%). We acknowledge that this may have been underestimated, as migraine with aura reportedly affects approximately 30% of patients with migraine [46]. The lower proportion of patients with migraine with aura in our study may be owing to the lower prevalence of migraine with aura in Asia [47, 48] and limitations on valid diagnosis of migraine based on insurance claim data compared to the clinic- or hospital-based diagnosis using structured questionnaires and clinical diagnoses. Third, clinical information, including information on prescribed medications, was not available to the researchers because of the inherent limitations of the NHIS database. However, anti-platelet [49, 50], cholesterol-lowering [51, 52], or calcitonin gene-related peptide-modulating medications (such as triptans) [53] might affect the development of VaD. Forth, confounding influences may exist between covariates, such as smoking and alcohol intake, which can affect comorbidities like hypertension and congestive heart failure. We adjusted for all covariates in Model 4 because those covariates have been considered as possible independent risks for dementia [54–56]. However, further studies are necessary to elucidate the effect of interactions between comorbidities and anthropometric characteristics on the risk of VaD. Fifth, inclusion of a limited number of participants with health examination data or exclusion of participants with missing data may have caused selection bias. Additionally, patients with migraine are more likely to visit clinics, which would increase the opportunities for dementia diagnosis. Finally, this study was conducted in the Korean population, and it is unclear whether the results can be generalized to non-Asian populations.

Overall, migraine was associated with an increased risk of VaD, and this association was greater in patients with chronic migraine. These finding may suggest that migraine should be considered as a modifiable risk factor to reduce the incidence of VaD.

## Supporting information

S1 Table ICD-10 codes for comorbidities. (DOC)

S2 Table Multivariable Cox proportional hazards regression analysis of the risk of vascular dementia in individuals with migraine with aura and those with migraine without aura. (DOC)

## Abbreviations

ASPREE: Aspirin in Reducing Events in the Elderly
BMI: Body mass index
CI: Confidence interval
HR: Hazard ratio
NHIS: National Health Insurance Service
SD: Standard deviation
