# Supplementary material for: Association between migraine and the risk of vascular dementia: A nationwide longitudinal study in South Korea
Source: PLoS One. 2024 Apr 17;19(4):e0300379. doi: 10.1371/journal.pone.0300379 (PMC11023172; doi:10.1371/journal.pone.0300379)
Supplement: S1 Table — (DOC) [file pone.0300379.s001.doc]

**S1 Table. ICD-10 codes for comorbidities**

| **Comorbidities** | **ICD-10 codes** |
| --- | --- |
| **Hypertension** | I10 (essential hypertension), I13 (hypertensive heart and renal disease), or I15 (secondary hypertension) |
| **Diabetes** | E11 (non-insulin-dependent diabetes mellitus), E12 (malnutrition-related diabetes mellitus), E13 (other specified diabetes mellitus), or E14 (unspecified diabetes mellitus) |
| **Dyslipidemia** | E78 (disorders of lipoprotein metabolism and other lipidemias) |
| **Myocardial infarction** | I21 (ST elevation and non-ST elevation myocardial infarction), I22 (subsequent ST elevation and non-ST elevation myocardial infarction), I23 (current complications following ST elevation and non-ST elevation myocardial infarction), I24 (other acute ischemic heart diseases), or I25 (chronic ischemic heart disease) |
| **Congestive heart failure** | I50.0 (congestive heart failure) |
| **Stroke** | I63 (cerebral infarction) or I64 (stroke, not specified as hemorrhage or infarction) |
| **Atrial fibrillation** | I48 (atrial fibrillation and flutter) |
| **Depression** | F32 (depressive episode) F33 (recurrent depressive disorder) |

Abbreviation: ICD-10, International Classification of Diseases, Tenth Revision
