# Supplementary material for: Association between migraine and the risk of vascular dementia: A nationwide longitudinal study in South Korea
Source: PLoS One. 2024 Apr 17;19(4):e0300379. doi: 10.1371/journal.pone.0300379 (PMC11023172; doi:10.1371/journal.pone.0300379)
Supplement: S2 Table — (DOC) [file pone.0300379.s002.doc]

**S2 Table. Multivariable Cox proportional hazards regression analysis of the risk of vascular dementia in individuals with migraine with aura and those with migraine without aura.**

|  |  | **Group** | **Participants (n)** | **VaD diagnosis (n)** | **Duration (Person-years)** | **Incidence rate per 1000 person-y** | **Model 3 ^a^, HR**  **(95% CI)** | **p for interaction** |  |
| --- | --- | --- | --- | --- | --- | --- | --- | --- | --- |
| **Age** | **Age <65** | Control | 4,821,001 | 16,966 | 44,383,172 | 0.382 | 1 (Ref.) | 0.041 |  |
|  |  | Without aura | 150,990 | 833 | 1,392,475 | 0.598 | 1.36 (1.27,1.46) |  |  |
|  |  | With aura | 7,250 | 33 | 66,991 | 0.493 | 1.18 (0.84,1.66) |  |  |
|  | **Age ≥65** | Control | 1,042,347 | 43,293 | 8,257,569 | 5.243 | 1 (Ref.) |  |  |
|  |  | Without aura | 52,527 | 2,924 | 402,313 | 7.268 | 1.23 (1.19,1.28) |  |  |
|  |  | With aura | 2,069 | 124 | 15,907 | 7.795 | 1.34 (1.13,1.60) |  |  |
| **Sex** | **Men** | Control | 3,128,807 | 29,694 | 27,924,658 | 1.063 | 1 (Ref.) | <0.001 |  |
|  |  | Without aura | 56,709 | 961 | 491,932 | 1.954 | 1.15 (1.08,1.22) |  |  |
|  |  | With aura | 2,361 | 35 | 20,657 | 1.694 | 1.01 (0.73,1.41) |  |  |
|  | **Women** | Control | 2,734,541 | 30,565 | 24,716,083 | 1.237 | 1 (Ref.) |  |  |
|  |  | Without aura | 146,808 | 2,796 | 1,302,855 | 2.146 | 1.31 (1.26,1.37) |  |  |
|  |  | With aura | 6,958 | 122 | 62,241 | 1.960 | 1.44 (1.20,1.71) |  |  |
| **Hypertension** | **No** | Control | 3,866,752 | 22,269 | 35,259,722 | 0.632 | 1 (Ref.) | 0.001 |  |
|  |  | Without aura | 117,008 | 1,240 | 1,059,148 | 1.171 | 1.39 (1.31,1.47) |  |  |
|  |  | With aura | 5,550 | 44 | 50,570 | 0.870 | 1.25 (0.93,1.68) |  |  |
|  | **Yes** | Control | 1,996,596 | 37,990 | 17,381,019 | 2.186 | 1 (Ref.) |  |  |
|  |  | Without aura | 86,509 | 2,517 | 735,639 | 3.422 | 1.21 (1.16,1.26) |  |  |
|  |  | With aura | 3,769 | 113 | 32,327 | 3.495 | 1.34 (1.11,1.61) |  |  |
| **Diabetes** | **No** | Control | 5,183,134 | 45,488 | 46,823,813 | 0.971 | 1 (Ref.) | 0.019 |  |
|  |  | Without aura | 180,801 | 2,938 | 1,608,475 | 1.827 | 1.30 (1.25,1.34) |  |  |
|  |  | With aura | 8,356 | 126 | 74,854 | 1.683 | 1.38 (1.16,1.65) |  |  |
|  | **Yes** | Control | 680,214 | 14,771 | 5,816,928 | 2.539 | 1 (Ref.) |  |  |
|  |  | Without aura | 22,716 | 819 | 186,312 | 4.396 | 1.17 (1.09,1.25) |  |  |
|  |  | With aura | 963 | 31 | 8,043 | 3.854 | 1.08 (0.76,1.53) |  |  |
| **Dyslipidemia** | **No** | Control | 4,624,529 | 42,516 | 41,617,455 | 1.022 | 1 (Ref.) | 0.014 |  |
|  |  | Without aura | 146,960 | 2,349 | 1,303,216 | 1.802 | 1.24 (1.19,1.29) |  |  |
|  |  | With aura | 6,741 | 84 | 60,377 | 1.391 | 1.11 (0.90,1.37) |  |  |
|  | **Yes** | Control | 1,238,819 | 17,743 | 11,023,286 | 1.610 | 1 (Ref.) |  |  |
|  |  | Without aura | 56,557 | 1,408 | 491,571 | 2.864 | 1.31 (1.24,1.38) |  |  |
|  |  | With aura | 2,578 | 73 | 22,520 | 3.242 | 1.66 (1.32,2.09) |  |  |
| **BMI** | **BMI <25** | Control | 3,822,407 | 39,390 | 34,228,948 | 1.151 | 1 (Ref.) | 0.790 |  |
|  |  | Without aura | 131,134 | 2,402 | 1,153,454 | 2.082 | 1.26 (1.21,1.31) |  |  |
|  |  | With aura | 6,068 | 99 | 54,023 | 1.833 | 1.27 (1.04,1.55) |  |  |
|  | **BMI ≥25** | Control | 2,040,941 | 20,869 | 18,411,793 | 1.133 | 1 (Ref.) |  |  |
|  |  | Without aura | 72,383 | 1,355 | 641,333 | 2.113 | 1.28 (1.21,1.35) |  |  |
|  |  | With aura | 3,251 | 58 | 28,875 | 2.009 | 1.38 (1.07,1.78) |  |  |
| **Current smoking** | **No** | Control | 4,549,585 | 49,305 | 40,910,769 | 1.205 | 1 (Ref.) | 0.023 |  |
|  |  | Without aura | 181,386 | 3,422 | 1,601,919 | 2.136 | 1.28 (1.24,1.33) |  |  |
|  |  | With aura | 8,419 | 146 | 74,988 | 1.947 | 1.36 (1.15,1.59) |  |  |
|  | **Yes** | Control | 1,313,763 | 10,954 | 11,729,972 | 0.934 | 1 (Ref.) |  |  |
|  |  | Without aura | 22,131 | 335 | 192,868 | 1.737 | 1.11 (1.00,1.24) |  |  |
|  |  | With aura | 900 | 11 | 7,910 | 1.391 | 0.92 (0.51,1.66) |  |  |

Abbreviations: BMI, body mass index; CI, confidence interval; eGFR, estimated glomerular filtration rate; HR, hazard ratio; VaD, vascular dementia.

^a^Adjusted for age, sex, comorbidities (hypertension, diabetes, dyslipidemia, myocardial infarction, congestive heart failure, and stroke), eGFR, BMI, and lifestyle (smoking status, drinking status, and regular exercise).
